# Insulin treatment guided by subcutaneous continuous glucose monitoring compared to frequent point-of-care measurement in critically ill patients: a randomized controlled trial

**DOI:** 10.1186/s13054-014-0453-9

**Published:** 2014-08-20

**Authors:** Daphne T Boom, Marjolein K Sechterberger, Saskia Rijkenberg, Susanne Kreder, Rob J Bosman, Jos PJ Wester, Ilse van Stijn, J Hans DeVries, Peter HJ van der Voort

**Affiliations:** Department of Intensive Care, Onze Lieve Vrouwe Gasthuis, PO Box 95500, Amsterdam, 1090 HM The Netherlands; Department of Internal Medicine, Academic Medical Center, University of Amsterdam, Meibergdreef 9, Amsterdam, 1105 AZ The Netherlands

## Abstract

**Introduction:**

Glucose measurement in intensive care medicine is performed intermittently with the risk of undetected hypoglycemia. The workload for the ICU nursing staff is substantial. Subcutaneous continuous glucose monitoring (CGM) systems are available and may be able to solve some of these issues in critically ill patients.

**Methods:**

In a randomized controlled design in a mixed ICU in a teaching hospital we compared the use of subcutaneous CGM with frequent point of care (POC) to guide insulin treatment. Adult critically ill patients with an expected stay of more than 24 hours and in need of insulin therapy were included. All patients received subcutaneous CGM. CGM data were blinded in the control group, whereas in the intervention group these data were used to feed a computerized glucose regulation algorithm. The same algorithm was used in the control group fed by intermittent POC glucose measurements. Safety was assessed with the incidence of severe hypoglycemia (<2.2 mmol/L), efficacy with the percentage time in target range (5.0 to 9.0 mmol/L). In addition, we assessed nursing workload and costs.

**Results:**

In this study, 87 patients were randomized to the intervention and 90 to the control group. CGM device failure resulted in 78 and 78 patients for analysis. The incidence of severe glycemia and percentage of time within target range was similar in both groups. A significant reduction in daily nursing workload for glucose control was found in the intervention group (17 versus 36 minutes; *P* <0.001). Mean daily costs per patient were significantly reduced with EUR 12 (95% CI −32 to −18, *P* = 0.02) in the intervention group.

**Conclusions:**

Subcutaneous CGM to guide insulin treatment in critically ill patients is as safe and effective as intermittent point-of-care measurements and reduces nursing workload and daily costs. A new algorithm designed for frequent measurements may lead to improved performance and should precede clinical implementation.

**Trial registration:**

Clinicaltrials.gov, NCT01526044. Registered 1 February 2012.

**Electronic supplementary material:**

The online version of this article (doi:10.1186/s13054-014-0453-9) contains supplementary material, which is available to authorized users.

## Introduction

Stress-induced hyperglycemia is common and relates to adverse outcomes in critically ill patients [1,2]. The outcomes of two large intervention studies are in some way contradictory but the consensus is that hyperglycemia should be corrected, while avoiding hypoglycemia and high glucose variability [3-8]. On the basis of the available evidence, it seems preferable to maintain a blood glucose level around 8.0 mmol/L for the majority of critically ill patients [9,10].

Glucose regulation regimens require frequent monitoring of glucose, which leads to a considerable workload for the intensive care (IC) nurses. In addition, glucose regulation carries an inherent risk of insulin-induced hypoglycemia, which is associated with mortality [6]. Information about the glucose level is lacking for the period in between measurements with possible unnoticed hypoglycemic episodes. Continuous glucose monitoring (CGM) could be of value to facilitate or improve glycemic control. Previous studies have indicated an acceptable accuracy and reliability for subcutaneous CGM devices in critically ill patients [11-15]. The only prospective randomized controlled trial so far that assessed the role for CGM in glycemic control in critically ill patients showed that real-time CGM increased the safety of tight glycemic control in critically ill patients by significantly reducing severe hypoglycemic events [16]. However, an improvement of the mean glucose concentration by using real-time CGM was not found [16].

Thus, CGM may give us the ability to detect early (possible) hypo- and hyperglycemia as well as minimizing swings in glucose levels. Moreover, the use of CGM may facilitate the process of glycemic control and may reduce the number of blood samples and accompanying blood loss, nursing workload and costs. To date, there are few data available how CGM-driven glucose regulation compares to point-of-care (POC) -driven glucose regulation and no controlled studies specifically evaluated workload and cost of CGM. The aim of the present study was to assess the safety, efficacy, workload and costs of a subcutaneous CGM system-guided blood glucose regulation in comparison with frequent POC blood glucose-guided regulation in a mixed population of critically ill patients.

## Material and methods

### Study design and participants

This was a randomized controlled open-label clinical trial, performed in a 20-bed mixed medical-surgical ICU of a teaching hospital (Onze Lieve Vrouwe Gasthuis, Amsterdam, the Netherlands). Patients were recruited over a period of 18 months from 2011 till late 2012. Patients were eligible for inclusion within 24 hours after ICU admission if they were 18 years or older, in need of intravenous (i.v.) insulin treatment for glucose regulation and with an expected length of stay in the ICU of at least 24 hours. Patients could not be included if any of the following criteria was present: lack of informed consent, participation in another trial or previous participation in this trial or when a CGM system was currently not available. The study ended when patients were discharged from the ICU or because of technical failure of the CGM device. The maximum study duration was set at five days for both treatment groups. The complete nursing staff was trained beforehand to handle all devices used in this study adequately. This study was approved by the ethics committee VCMO, Nieuwegein, The Netherlands and was in line with Dutch and European legislation. All patients or their legal representative provided written informed consent. This trial is registered with Clinicaltrials.gov, number NCT01526044.

### Randomization

Patients who met the inclusion criteria were randomized in a 1:1 ratio with computerized block randomization to either the intervention group or the control group.

### Study procedures

#### Algorithm

In all study participants, blood glucose regulation was performed by a sliding scale algorithm with a blood glucose target of 5.0 to 9.0 mmol/L, which was integrated into the patient data management system (PDMS, MetaVision; *i*MD*soft*, Tel Aviv, Israel) [17]. Hypoglycemia was defined as a blood glucose level of <2.2 mmol/L in line with the Van den Berghe trial [3]. Below target was defined as a glucose level from 2.2 mmol/L till the lower target level of 5.0 mmol/L. Above target, all glucose levels were above 9.0 mmol/L. The algorithm instructed the insulin i.v. infusion rate (or glucose administration in case of hypoglycemia) after each glucose measurement. The time for the next glucose measurement was also defined from the algorithm and depended on the stability of the glucose level over time.

#### Glucose measurement

Study participants allocated to the intervention group received a subcutaneous CGM system (FreeStyle Navigator™, Abbott Diabetes Care, Alameda, CA, USA), which was used to guide blood glucose regulation. The nurses were trained to insert the subcutaneous glucose sensors on the patients’ abdomen or upper arm. After insertion of the subcutaneous sensor, a transmitter was attached that connects through wireless communication to a receiver, which displays the real-time glucose readings every minute and stores glucose readings every 10th minute. The CGM system needed a one-hour stabilization period, in which glucose measurements were not performed. Calibration of the system using an arterial blood sample was performed five times in total, after 1, 2, 8 to 10, 24 to 32 and 72 to 80 hours, following manufacturer instructions. The CGM system sounded an alarm when additional calibrations were needed. On the times that the algorithm needed a new glucose measurement, the readings from the CGM system were entered in the computerized glucose regulation protocol that was embedded in the PDMS. Other CGM values were not used in the algorithm. The CGM system sounded an alarm when the glucose level was either <5.0 mmol/L or >9.0 mmol/L. When this occurred, the nurse entered this additional glucose level in the computerized protocol, which triggered the glucose algorithm to advise an insulin dosing adjustment. The CGM repeated its alarm after 15 minutes when the glucose level was still out of target range. Again, this value was entered into the system and dose adjustments were made until target range was achieved. Every hypoglycemic event (<2.2 mmol/L) needed to be verified by an arterial blood glucose sample. In case of a discrepancy between the CGM value and the arterial blood glucose sample, the latter was leading in clinical decision-making.

Blood glucose regulation in the study participants allocated to the control group was performed by use of frequent point-of-care (POC) measurements using Accu-Chek™ (Roche/Hitachi, Basel, Switzerland). All blood samples were obtained from an indwelling arterial catheter. The displayed glucose levels were automatically stored in the PDMS. Participants in the control group also received a subcutaneous Freestyle Navigator CGM system, however, these data were blinded and not used for blood glucose regulation. Calibrations were performed following manufacturer instructions and no alarms were set.

In both groups arterial reference blood glucose samples were drawn six times daily at standardized times and analyzed by the ABL Flex automated blood gas analyzer (BGA) (Radiometer, Copenhagen, Denmark). These values were automatically stored into the PDMS but were blinded to both nurses and physicians.

#### Study endpoints

The primary safety outcome was the incidence of severe hypoglycemia (<2.2 mmol/L) during the intervention. Efficacy outcomes were the percentage of time that glucose levels were within the target range (5.0 to 9.0 mmol/L), below target range (2.2 to 5.0 mmol/L), and in the hyperglycemic range (>9.0 mmol/L). In addition, mean blood and sensor glucose levels and glucose variability defined as the mean absolute glucose (MAG) change (ΔGlucose/ΔTime) were endpoints too [8]. The accuracy of the CGM and the POC device was assessed by calculating the median relative absolute deviation (RAD) between reference glucose and CGM or POC glucose.

Nursing workload for glucose control per day was determined by the number of POC measurements or measurements from the sensor, which were entered in the computerized glucose regulation protocol and the amount of calibrations of the CGM sensor (in the intervention group only). A time-in-motion design was used to estimate the time that it took to execute targeted glucose control and insulin treatment per group. The following subtasks were observed: (1) POC measurement (this included the initiation, blood sampling, blood testing and processing), (2) sensor placement, (3) sensor calibration and (4) time needed to determine a CGM value and entering the value in the decision support module. The tenfold-recorded elapsed times per subtask were averaged and then multiplied by the 24-hour blood sample average collected from the clinical trial.

Cost analysis was performed from a health-care payer perspective with a one-day (24 hours) time horizon. The outcome measure in the economic evaluation was the costs per patient for glycemic control in 24 hours. Cost parameters included nursing personnel costs, device costs, materials needed for glucose monitoring and laboratory costs. Cost estimates for the parameters were derived from the hospital and laboratory ledger, devices manufacturers’ data and the Dutch guide for health economic research [18]. Costs are expressed in euros and are based on the year 2013. Because of the short time horizon of this analysis (24 hours), the costs were not discounted.

### Data collection

Clinical and laboratory baseline data were extracted from the PDMS after randomization: demographic data, body mass index (BMI), reason for ICU admission, history of diabetes, history of renal failure, severity of disease scores (the sequential organ failure assessment (SOFA) score and acute physiology and chronic health evaluation (APACHE IV) score at admission), blood glucose levels at admission and the use of mechanical ventilation. Blood glucose data, that is reference arterial blood glucose samples and glucose values that were entered in the decision support module (CGM measurements in the intervention group, POC measurements in the control group) were also extracted from the PDMS. Continuous glucose data from the CGM device were uploaded to a computer using CoPilot™ Health Management System for FreeStyle Navigator (Abbott Diabetes Care, Alameda, CA, USA) and entered in the study database. All reference glucose measurements were linked by time with the concomitant CGM measurements and Accu-Chek measurements.

### Statistical analysis

A sample size of 160 (80 participants in each group) conferred 80% power, with two-sided *P* = 0.05, to detect an absolute difference of 10% in the incidence of severe hypo- or hyperglycemia between the intervention and the control group. A total sample size of 178 patients (89 patients per group) is needed to correct for an expected 10% drop out. Results are expressed as percentages for categorical variables, mean and standard deviation (SD) for continuous normally distributed variables, and median and interquartile range (IQR) for continuous non-normally distributed variables. Groups were compared by using Fisher’s exact test, Student’s *t* test or Mann-Whitney rank-sum test as appropriate. Median RAD was calculated instead of mean because of its skewed distribution. Costs were calculated as the summed product of factors and resources used and their respective unit costs and were averaged per patient per day. Because of skewed (cost) distributions, we assessed group contrasts by calculating 95% confidence intervals for the mean differences following bias-corrected and accelerated nonparametric bootstrapping, that is drawing 1,000 samples of the same size as the original sample separately for each group. All statistical analyses were performed in SPSS 20.0 (IBM Corp, Armonk, NY, USA).

## Results

**Figure 1:**
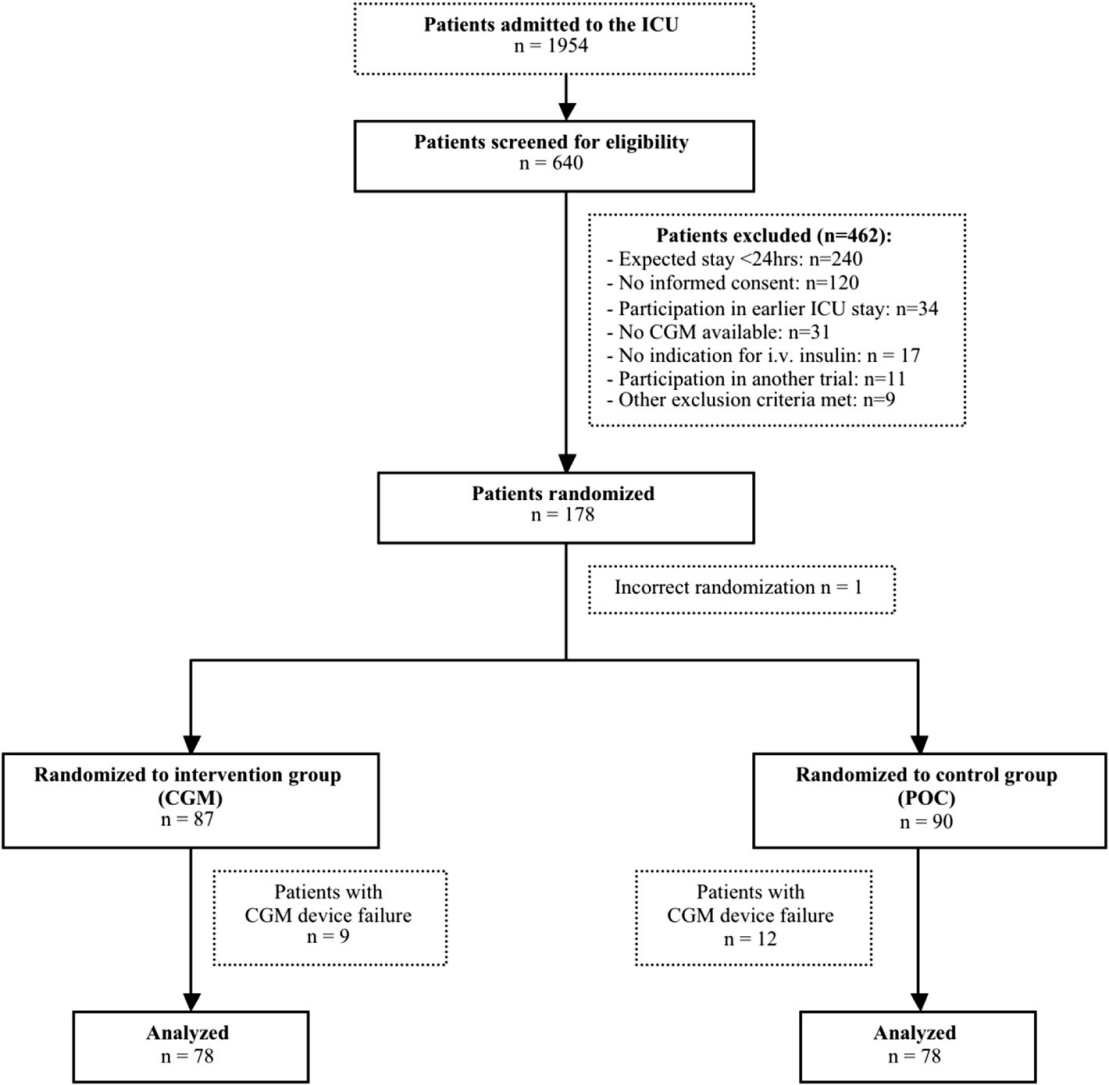
**Flow chart of study participants: assessment, randomization and analysis.**

A total of 178 patients were randomized to either the intervention or the control group (Figure 1). Most of the patients who were not eligible were postoperative cardiac surgery patients with an expected length of stay (LOS) <24 hours. One patient was incorrectly randomized and did not receive a CGM device. Nine patients in the intervention group and twelve patients in the control group were excluded from analysis due to lack of CGM data because of technical failure of the device, misplacement of the sensor (n = 3) and problems with extraction of the data (n = 18).

We performed a per protocol analysis from the data of 78 patients in each group. Table 1 shows the two groups, which were well matched with respect to all baseline characteristics.

**Table 1:** **Baseline characteristics of participants**

	**Intervention - CGM**	**Control - POCM**
	**(** ***n*** **= 87)**	**(** ***n*** **= 90)**
Age (years)	664 (14.0)	67.2 (11.4)
Women	45 (52%)	35 (39%)
BMI (kg/m^2^)	27.8 (7.0)	27.4 (5.8)
Weight (kg)	81.8 (21.7)	83.2 (21.5)
History of diabetes^*^	18 (21%)	21 (23%)
History of renal failure^**^	10 (12%)	5 (6%)
Reason for ICU admission		
Surgical		
Elective	19 (22%)	16 (18%)
Emergency	12 (14%)	13 (14%)
Medical	56 (64%)	61 (68%)
Admission diagnosis		
Post cardiac surgery	12 (14%)	11 (12%)
Severe sepsis/septic shock	23 (26%)	18 (20%)
Pneumonia	12 (14%)	11 (12%)
Cardiac failure	10 (12%)	9 (10%)
COPD	3 (3%)	8 (9%)
Hemorrhagic shock	7 (8%)	10 (11%)
Cardiac arrest/resuscitation	10 (12%)	14(16%)
Other	10 (12%)	9 (10%)
APACHE IV predicted mortality (%)	32 (10-70)	31 (20-60)
SOFA score on admission	8 (6-10)	7 (6-10)
Blood glucose level on admission (mmol/L)	9.0 (2.6)	9.2 (2.5)
Mechanical ventilation	80 (92%)	83 (92%)

Data are mean (SD), median (IQR) or n (%). ^*^Diabetes was defined as present when this diagnosis was mentioned in the medical history; ^**^renal failure was present when the preadmission serum creatinine was above 177umol/l. CGM: continuous glucose monitoring, POCM: point-of-care monitoring, BMI: body mass index, ICU: intensive care unit, SOFA: sequential organ failure assessment, COPD: chronic obstructive pulmonary disease; APACHE: acute physiology and chronic health evaluation.

During the intervention, a total of 37,570 (intervention group) and 32,957 (control group) CGM measurements were collected. The number of reference arterial blood gas glucose measurements was 1,599 in the intervention group and 1,325 in the control group. The median number of additional calibrations needed for the CGM was 1.9 per 24 hours (IQR 1.2 to 3.3). The number of glucose values entered in the PDMS (CGM measurements in the intervention group and POC measurements in the control group) was 3,919 and 2,489 respectively.

Table 2 summarizes the outcome measures of the study. The incidence of hypoglycemia (<2.2 mmol/L), the primary safety endpoint, was similar in both the intervention and the control group. None of the severe hypoglycemic episodes detected by the CGM in the intervention group was verified by arterial blood sampling. In the control group, all severe hypoglycemic episodes detected by the CGM, occurred in between two POC glucose measurements and were not detected by the nurses. In total, there were 14 patients (3 patients in the control group and 11 patients in the intervention group) who experienced 19 ‘true’ hypoglycemic events (<3.9 mmol/L) detected by ABL. Twenty-five percent (n = 4) of the true ‘hypoglycemic’ events in the CGM group and 67% (n = 2) in the control group were also identified by CGM or POC (difference in glucose ≤10%). All other endpoints such as percentage time in target range, below target range, mean reference and sensor glucose, glucose variability, hospital LOS, ICU and hospital mortality were nonsignificantly different between the study groups. Moderate hyperglycemia (9.0 to 11.1 mmol/L) was significantly different in favor of the intervention group (*P* = 0.03). A total of 355 time-linked reference glucose CGM samples and 85 time-linked reference glucose POC samples were used to assess accuracy of the devices. Median (IQR) RAD of the POC device was 7.1% (3 to 12) whereas the median RAD of the CGM device was 13.7% (8 to 23) (*P* <0.001). Bland-Altman plots per glucose monitoring system are shown in an additional file (Figure S1 in Additional file 1).

**Table 2:** **Safety, efficacy and clinical study outcomes**

	**Intervention - CGM**	**Control - POCM**	***P***
	**(** ***n*** **= 78)**	**(** ***n*** **= 78)**	
Study period (days)	3.2 (2-5)	2.8 (1-5)	0.18
Incidence severe hypoglycemia (<2.2 mmol/L)^1^	None	None	
Detected by CGM			
Number of subjects	3 (4%)	4 (5%)	1.0
Episodes < 2.2 mmol/L	3	4	
% of time for the reference glucose level (SD)^3^			
In target range (5.0-9.0 mmol/L)	69 (26)	66 (26)	0.47
Below target range (2.2-5.0 mmol/L)	5 (7)	3 (5)	0.21
Mild/moderate hypoglycemia (2.2-3.9)	1 (3)	0 (1)	0.03
Above target range (>9.0 mmol/L)	28 (26)	34 (27)	0.06
Mild/moderate hyperglycemia (9.0-11.1)	17 (16)	26 (23)	0.01
Hyperglycemia (>11.1)	11(19)	7(14)	0.19
% of time for the sensor glucose levels (SD)^3^			
In target range (5.0-9.0 mmol/L)	75 (18)	71 (20)	0.18
Below target range (2.2-5.0 mmol/L)	11 (13)	9 (12)	0.44
Mild/moderate hypoglycemia (2.2-3.9)	2 (7)	1 (2)	0.14
Above target range (>9.0 mmol/L)	15 (16)	20 (21)	0.06
Mild/moderate hyperglycemia (9.0-11.1)	12 (11)	16 (16)	0.03
Hyperglycemia (>11.1)	3 (7)	4 (9)	0.35
Mean reference blood glucose (mmol/L)	8.2 (1.6)	8.3 (1.3)	0.53
Mean sensor glucose (mmol/L)	7.1 (1.1)	7.5 (1.3)	0.07
MAG change (mmol/L/h)^2^	0.33 (0.2-0.5)	0.32(0.2-0.4)	0.31
LOS ICU (hours)	137 (71-250)	95 (51-157)	0.04
LOS hospital (days)	15 (8-270)	14 (8-31)	0.91
Mortality ICU	15 (19%)	12 (15%)	0.67
Mortality hospital	22 (28%)	17 (22%)	0.46

Data shown are mean (SD), median (IQR), or *n* (%). ^1^Patients who experienced at least one severe hypo- or hyperglycemic episode, verified by blood gas analysis; ^2^when at least three reference glucose measurements were available (intervention *n* = 73, control *n* = 71); ^3^percentages do not add up to 100 due to rounding off. CGM: continuous glucose monitoring; POCM: point-of-care measurement, MAG: mean absolute glucose change; LOS: length of stay; ICU: intensive care unit.

Table 3 summarizes nursing workload data per 24 hours. The first column displays the average time burden per subtask of glucose control. The average total time burden for glucose control was significantly lower in the intervention group compared to the control group (17 minutes versus 36 minutes; *P* <0.001). The mean reduction in total nursing workload was 19 minutes per 24 hours or 53% in favour of the intervention group. As in this study, an open blood drawing system was used, 5 mL blood per POC measurement or calibration was taken from the patient. Blood loss was therefore significantly reduced in the intervention group (15.3 mL versus 60 mL per day; *P* <0.001).

**Table 3:** **Nursing workload per day (24 hours)**

	**Time per action (min)**	**Number of actions in control group**	**Nursing time control group (min)**	**Number of actions in intervention group**	**Nursing time intervention group (min)**
POC measurement	3	12 (8)	36 (24)	0.06 (0.4)	0.2 (0.4)
Sensor CGM placement	3.5	-	-	1	3.5
Sensor CGM calibration	2.5	-	-	1.9 (1.2-3.3)	8 (11)
Sensor CGM data to enter in PDMS	0.3	-	-	18 (10)	5.3 (3)
**Total time (min)**			**36 (24)**		17 (12)*

Data are expressed as mean (SD), or median (IQR). *P <0.001 in comparison with control group. POC: point-of-care; CGM: continuous glucose monitoring; PDMS: patient data management system.

The economic analysis of both groups is shown in Table 4. The intervention group generated an average total daily cost of EUR 41, whereas the total daily cost in the control group was EUR 53. The difference in costs was EUR −12 in favor of the intervention group (95% CI −32 to −18, *P* = 0.02). The extra costs of the CGM devices in the intervention group were neutralized by the diminished costs for nursing personnel, material and laboratory costs.

**Table 4:** **Cost analysis**

	**Costs per unit**	**Factor control group**	**Costs in control group**	**Factor intervention group**	**Costs in intervention group**	**Difference in costs (95% ** **CI)** ^**1**^
Nursing time	€38/hr	36 min	€22.98	17 min	€10.87	€-12.11(−16, −9)
CGM receiver	€1009.59	-	-	€1.38 per day^2^	€1.38	€1.38
CGM sensor	€61.00	-	-	€24.40 per day^3^	€24.40	€24.40
CGM calibration^4^	€1.19	-	-	3.3	€3.95	€3.95 (3,5)
Accu-Chek Inform II device	€892.37	€1.22 per day^2^	€1.22	-	-	€-1.22
Material POC measurement^5^	€0.70	12.2	€8.51	0.06	€0.04	€-8.47 (−10, −7)
Laboratory^6^	€1.66	12.2	€20.18	0.06	€0.10	€-20.08 (−23, −18)
**Total costs**			**€52.89**		**€40.74**	**€-12.42 (−22, −5)**

Factors and costs are expressed as means per patient per day (24 hours). ^1^95% confidence interval based on 1,000 stratified bootstrap samples; ^2^assuming a lifetime of two years; ^3^assuming a manufacturers’ sensor lifetime of two and a half days; ^4^calibration strip CGM; ^5^includes syringes, nonsterile gloves, gauzes, alcohol, cap (used for blood sampling) and testing strip POC; ^6^costs for a single point-of-care glucose measurement. CGM: continuous glucose monitoring, POC: point-of-care.

## Discussion

The present study showed that a subcutaneous CGM system to guide blood glucose regulation was equally effective and safe in glycemic control compared to frequent POC-guided blood glucose regulation. However, CGM significantly reduces nursing workload, blood loss and the daily costs for glucose control.

### Comparison with other studies

This is the second but largest randomized controlled trial in which CGM is used to guide glycemic control in critically ill patients. In contrast to our findings, Holzinger and colleagues did find less severe hypoglycemia in the CGM group [16]. This may be caused by the very low incidence of severe hypoglycemia in the present study, which was true for both the intervention and the control group. This may be related to a change of policy after the publication of the NICE-SUGAR trial [4], which was a reason for our and most other ICUs to increase their blood glucose target range. The increased target range may have reduced the incidence of hypoglycemic events [19,20]. Indeed, the blood glucose target used in the current study (5.0 to 9.0 mmol/L) was higher than in the Holzinger trial [16] (4.4 to 6.1 mmol/L) and this is reflected in the achieved mean blood glucose levels (8.1 vs. 6.3 mmol/L). Moreover, the use of a fully computerized algorithm for glucose control and the high familiarity of the protocol among our IC nurses may have contributed to the low incidence of severe hypoglycemia. The available studies to date on tight glucose control showed an increase in nursing workload [21-23]. The potential benefits of CGM in the reduction of blood samples, blood loss and nursing workload was assumed in previous studies, but was not systematically assessed before. We now observed that CGM significantly reduced the amount of blood samples and the daily nursing workload for glucose control up to 53%. This finding seems clinically relevant, especially in a busy clinical IC environment. Two studies focused on the cumulative nursing workload accompanied with tight glucose control protocols [21,22]. Gartemann *et al*. estimated that nurses devoted approximately 42 minutes during a 12-hour shift of their time to administering a tight glycemic control (TGC) protocol, whereas Aragon *et al*. even reported that up to 2 hours might be required for tight glycemic control for a single patient in a 24-hour period. In our POC control group, the mean nursing workload estimate was less (36 minutes per 24 hours) than the published estimates reported by other groups. This might partly be explained by the use of a fully computerized algorithm for glucose control in our ICU. In addition, the familiarity of the protocol is very high among our ICU nurses.

### Effectiveness and costs

The use of CGM did not achieve improved glycemic control in our study. We found similar percentages of time-in-target and below-target range between the study groups. The not-significantly lower percentage of time in the hyperglycemic range in the intervention group could be explained by the fact that CGM measurements were more frequently entered in the glucose protocol than POC measurements in the control group. This probably resulted in more adjustments in the insulin treatment with lower blood glucose levels as a consequence. The significantly increased ICU LOS, which was observed in the intervention group, may be a coincidence or reflect unmeasured case-mix factors but is, in our view, unrelated to the glucose measurement strategy.

In contrast to our expectations, the cost analysis shows that the use of CGM systems for glucose control in an ICU setting is not *a priori* an expense. However, we should be cautious in interpreting these results due to the rather short time horizon (24 hours) in the analysis of costs determination and the single-centre study design. Also, cost savings cannot immediately be monetized due to the short time horizon used in this cost analysis.

### Accuracy of the subcutaneous measurements

The subcutaneous Freestyle Navigator CGM device that we used in the present study showed a median RAD of 13.7%, which is higher than the 10.6 and 11.6% that was found in previous validation studies of this device in critically ill patients, suggesting an accuracy acceptable for clinical use. [11,14]. The lag time that may be needed for the subcutaneous compartment to adapt to the intravenous compartment appeared not to be clinically relevant [11]. However, the accuracy as assessed in the current study seems to indicate a need for improvement, because the accuracy was less than the accuracy of the Accu-Chek and because a substantial number (75% in the CGM group and 33% in the control group) of hypoglycemic events was not detected. Of note, Leelarathna *et al*. [24] recently investigated whether there was a difference in accuracy of the Freestyle Navigator in a critical care setting using two methods of calibration: (1) calibration according to the manufacturer’s instructions (1, 2, 10, and 24 h) or (2) calibration at variable intervals of 1 to 6 h using ABG. Using enhanced calibration, at a median (interquartile range) every 169 (122 to 213) minutes, the absolute relative deviation was lower (7.0% (3.5, 13.0) vs. 12.8% (6.3, 21.8), *P* <0.001). So, further significant improvements in accuracy may be obtained by frequent calibrations with ABG measurements. In the current study forced calibration was not possible, calibration was only performed when the CGM device indicated the need for calibration by itself.

In addition, technical problems with the subcutaneous CGM device were observed during the study and led to a 12% dropout. The most important reason was the temporary loss of sensor signal from several minutes to hours that resulted in a loss of data. Difficulties in the calibration process were also identified as the CGM could only be calibrated if the system indicated a calibration by itself, which occurred for median 1.9 times per 24 hours. Most of the technical difficulties, however, may have been due to lack of experience working with the CGM device despite the training of all ICU nurses. We expect such problems to be easily resolved with additional training and with the improved next generation Freestyle Navigator II, which has recently been introduced and showed good utility and sensor performance in critically ill patients [25]. This study aimed to define safety, efficacy and costs and therefore we neglected the system dropout at this moment. It is true, however, that this device can only become part of routine care when the dropout percentage diminishes.

### Strengths and weaknesses

The strengths of our study include the relatively large sample size, the randomised controlled study design and the wide variety in case mix. However, some limitations of the present study merit further consideration. First, the study was performed in a single Dutch intensive care unit, which limits the generalizability of the study. Second, the study was designed to blind the values of the CGM in the control group. However, the CGM needed to be calibrated several times during the study period, which made it impossible to blind it completely. Third, the nursing staff did not verify the severe hypoglycemia that was indicated by CGM in two of the three patients despite specific instructions to do so. One of these two patients had evolved into a ‘withholding care policy’, which was the reason to accept the severe hypoglycemia. We assume that in the other patient priority was given to other important nursing tasks. Thus, the available data are insufficient to define the accuracy of the CGM in the hypoglycemic range. In our previous studies this was not identified as a clinical problem [11,14]. Also, with an adapted algorithm, the CGM should be able to detect a decreasing glucose level before hypoglycemia is present and give a timely alert. Fourth, the computerized algorithm was designed for intermittent POC measurements and not for (semi-) continuous data. As such, the patients did not fully benefit from the frequent glucose measurements by CGM. An algorithm based on 10-minute glucose input might have led to other results. We did identify this issue beforehand but we decided to keep the algorithm for both groups the same to be able to investigate the contribution of CGM *per se*. It can be expected that an adapted algorithm will further improve the performance of CGM in the guidance of glycemic control.

## Conclusions

Subcutaneous CGM to guide blood glucose regulation in critically ill patients was shown to be safe in terms of hypoglycemia incidence. With an identical insulin treatment algorithm, the CGM was equally effective as POC measurement. A new algorithm designed for frequent measurements may further improve the results and should precede clinical implementation. CGM significantly reduced nursing workload, blood loss and the daily costs for glucose control.

## Key messages

- Insulin treatment based on continuous subcutaneous glucose monitoring (CGM) revealed the same number of hypoglycemic events compared to point of care (POC)
- Subcutaneous CGM was equally effective as POC measured as glucose time in target range
- Total costs were lower when using subcutaneous CGM than frequent POC
- Nursing workload with glucose regulation was reduced by subcutaneous CGM compared to frequent POC
- A new algorithm designed for continuous measurement should be developed before CGM can be implemented clinically

## Additional file

Additional file 1: Figure S1. Bland-Altman plots per glucose monitoring system **(A)**, CGM system (Freestyle Navigator) **(B)**, point-of-care measurement (Accu-Chek). The x-axis represents the average of the sensor or device and reference glucose values in mmol/l. The y-axis represents the absolute difference between sensor or device and reference glucose values in mmol/l. The dotted lines represent the 5th and 95th percentile.
