# Supplementary figures and images for: Insulin treatment guided by subcutaneous continuous glucose monitoring compared to frequent point-of-care measurement in critically ill patients: a randomized controlled trial
Source: Crit Care. 2014 Aug 20;18(4):453. doi: 10.1186/s13054-014-0453-9 (PMC4161875; doi:10.1186/s13054-014-0453-9)

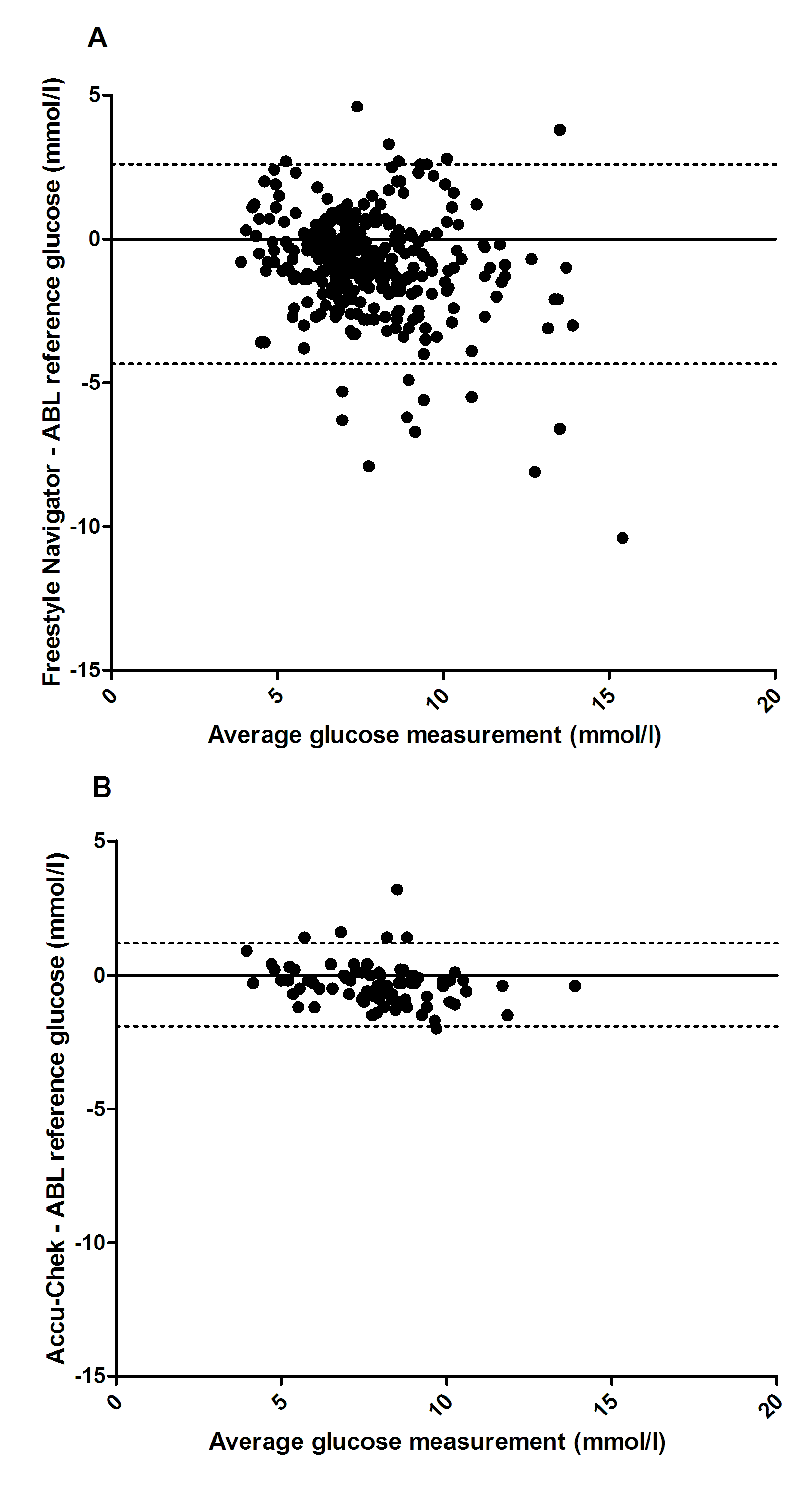

Supplement: Additional file 1: Figure S1. — Bland-Altman plots per glucose monitoring system (A), CGM system (Freestyle Navigator) (B), point-of-care measurement (Accu-Chek). The x-axis represents the average of the sensor or device and reference glucose values in mmol/l. The y-axis represents the absolute difference between sensor or device and reference glucose values in mmol/l. The dotted lines represent the 5th and 95th percentile. [file 13054_2014_453_MOESM1_ESM.tiff]
